# NK Cells: A Powerful Squad Versus SARS-CoV-2

**DOI:** 10.3390/ijms26136500

**Published:** 2025-07-06

**Authors:** Diana Lorena Alvarado-Hernández, Marlen Vitales Noyola, Ricardo Martínez-Rider, Sofía Bernal-Silva, Andreu Comas-Garcia

**Affiliations:** 1 Laboratory of Chemistry, Physics and Math Department, Autonomous University of San Luis Potosí, San Luis Potosí 78210, Mexico; diana.alvarado@uaslp.mx; 2 School of Medicine, Universidad Cuauhtémoc San Luis Potosí, San Luis Potosí 78290, Mexico; 3 Endodontics Postgraduate Program, Faculty of Dentistry, Autonomous University of San Luis Potosí, San Luis Potosí 78210, Mexico; marlen.vitales@uaslp.mx; 4 Department of Molecular Biomedicine, Center of Research and Advanced Studies of the National Polytechnical Institute (CINVESTAV), Mexico City 07360, Mexico; 5 Oral and Maxillofacial Surgery Specialty, Faculty of Dentistry, Autonomous University of San Luis Potosí, San Luis Potosi 78210, Mexico; rmrider@uaslp.mx; 6 Department of Microbiology, School of Medicine, Autonomous University of San Luis Potosí, San Luis Potosí 78210, Mexico; sofia.bernal@uaslp.mx

**Keywords:** NK cells, receptors, KIR, lectin-type, SARS-CoV-2

## Abstract

The function of NK cells in cancer and viral infections is well documented and understood. NK cell activity, including cytokine secretion, cytotoxic activity, and the coordination of inhibitory and activating receptors, linking innate and adaptive immunity, among others, has been examined for numerous pathogens, including parasites, bacteria, and viruses. The emergence of the SARS-CoV-2 health crisis has exposed a deficiency in understanding the previously elucidated mechanisms; the rationale for the reported variability in symptomatology among COVID-19 patients is extensive and intricate. It is evident that NK cells exert a significant influence on symptom severity, and their absence, with the presence or absence of their surface receptors, elicits a tailored response to the virus. This overview examines the impact of NK cells on the progression of several viral diseases, emphasizing their involvement in the pathogenesis of SARS-CoV-2 via the activation of surface receptors.

## 1. Introduction

Natural killer (NK) cells are a subset of granular lymphocytes that play a pivotal role in innate immunity. They are found in the blood (15% of immune cells) and in tissues like the skin and liver, where they can make up to 50% of immune cells. NK cells are characterized by their ability to both produce cytokines and exert cytotoxic effects against infected or abnormal cells, making them essential components of the body’s defense against pathogens and cancer [1,2].

Since their discovery in the 1970s, natural killer (NK) cells have been the subject of countless discoveries concerning their biology, interactions, structure, molecular mechanisms, epigenetics, biological roles, and potential therapeutic applications [3,4]. Their role in maintaining the innate immune system relies on their ability to secrete cytokines and exert cytotoxicity, as well as their regulatory functions in supporting the proper functioning of T cells and antigen-presenting cells, such as dendritic cells and macrophages [5].

The intricate role NK cells play in immune surveillance—demonstrated by their involvement in tumor growth regulation, allograft rejection, implantation processes, and their indispensable antiviral activity against herpesviruses, human papillomavirus, and fungal pathogens—gains even greater significance when examined within the historical context of global infectious diseases [2,6,7,8,9]. Humanity has been recurrently challenged by epidemic outbreaks and, at times, pandemics, phenomena distinguished by both the scale and dissemination pattern of pathogenic agents [10].

An *epidemic* is defined as the occurrence of disease within a specific community or region at a frequency in excess of the normal expectancy, marked by a substantial rise in case numbers over a defined period and geographic area. In contrast, a pandemic refers to an epidemic that has extended across multiple continents or globally, affecting a significant proportion of the world’s population. Historical examples—from the Black Death to the 1918 Spanish influenza—have underscored the capacity of certain pathogens to disrupt public health on an unprecedented scale. The relatively recent emergence of SARS-CoV-2, which precipitated a global pandemic, serves as a contemporary illustration of how viral agents can trigger devastating immunopathological responses [10]. During SARS-CoV-2 pathogenesis, as will be later addressed in greater detail, a particularly critical event—termed the cytokine storm—was documented in patients with fatal outcomes. This condition is characterized by a hyperactivation of the innate immune system, culminating in the uncontrolled and excessive release of proinflammatory mediators. These mediators, in concert with other immune cells, contribute to the development of multiorgan failure [3]. Notably, NK cells exhibit unique functional attributes, including the ability to generate antigen-specific immune memory without altering their receptor repertoire during maturation, establishing them as a cell type of paramount importance in the orchestration of immune responses [11,12].

Initially recognized for their ability to regulate tumor growth, numerous clinical trials have extensively studied and employed NK cells in anti-tumor strategies. Additionally, NK cells are crucial for rejecting tissues from other people and are key in the process of implantation while ensuring the body does not attack its cells. Their antiviral activity, perhaps the most studied aspect of NK cell function, is particularly critical in patients with congenital deficiencies or a complete absence of NK cells, who are highly susceptible to infections, especially those caused by herpesviruses. Nonetheless, infections from human papillomavirus and other fungal pathogens have also been documented, highlighting NK cells’ crucial role in combating viral infections [8,13,14,15,16].

During the pathogenesis of SARS-CoV-2, which is discussed in more detail later, patients with fatal outcomes experienced a specific event initially described as the cytokine storm. This condition involves the overactivation of the innate immune system, leading to an excessive and uncontrolled release of inflammatory molecules, particularly IL-1b, IL-6, IL-8, IL-12, IL-18, and TNF-a at the beginning of the disease and others like CXCL10, GDF15, TNSF1, and PTX4 in the recovery phase. These molecules, along with other immune cells, contribute to multiorgan failure. Notably, NK cells have special abilities, like creating immune memory for specific antigens without changing their receptors as they mature, which makes them a very important type of cell [3,17,18].

In this review, an analysis of the activation, phenotypic alterations, and functional dynamics of NK cells during SARS-CoV-2 is presented, elucidating their dualistic role in antiviral defense and their potential contribution to immunopathology. We present evidence of the diverse and sometimes contradictory roles of NK cells in the pathogenesis of infectious diseases, particularly emphasizing their significant involvement in SARS-CoV-2 infection.

## 2. Briefly, Where Do They Come from?

Initially, it was widely believed that the bone marrow was the exclusive site where NK cells originate, develop, and mature. This classical view positioned the bone marrow as the central organ for NK cell ontogeny, where hematopoietic stem cells (HSCs) are the progenitors of the NK cells, which undergo sequential stages of differentiation and maturation before entering the circulation as fully functional NK cells. However, recent advances in immunology have changed their understanding. Recent research has demonstrated that NK cell development is a more complex and distributed process, occurring not only in the bone marrow but also in several other tissues and organs. These include the thymus, secondary lymphoid tissues (such as lymph nodes and spleen), umbilical cord blood, peripheral blood, colon, uterus, kidney, gut, liver, and lungs. In these sites, NK cells can undergo local maturation and acquire tissue-specific phenotypic and functional characteristics that are finely tuned to the requirements of their microenvironment [19,20,21,22].

For example, the thymus has been shown to support the development of a distinct subset of NK cells with unique functional properties, while the uterus is home to uterine NK (uNK) cells that play a crucial role in pregnancy and placental development. Similarly, the liver harbors a specialized population of tissue-resident NK cells that contribute to immune surveillance and tissue homeostasis, and the lungs and colon contain NK cells adapted to mucosal immunity and local defense against pathogens [23,24,25].

These tissue-resident NK cells often differ markedly from their circulating counterparts in terms of surface marker expression, cytokine production, cytotoxic potential, and longevity. For instance, liver-resident NK cells express high levels of CXCR6 and display a memory-like phenotype (without the capacity to rearrange their receptor), while uterine NK cells are characterized by high expression of CD56 and unique functional roles in vascular remodeling. The recognition that NK cell development and specialization occur in multiple organs has important implications for our understanding of immune surveillance, tissue homeostasis, and the design of NK cell-based immunotherapies [21,23,25].

All the different types of NK cells come from a common starting point, the multipotent hematopoietic stem cell, which has specific traits (CD34+/CD10−/CD38LOW/CD45RA−), and this leads to a common lymphoid progenitor that can develop into different NK cell types. Some authors mentioned that the main subsets of NK cells are CD56^bright^ and CD56dim, but there are other relevant subsets. But probably the three main subsets are as follows: (a) The NK cells with a CD56^bright^/NKp80+/CD16−/CD94^high^/CD62L+ phenotype, which can be found in tissues or circulation, are capable of producing cytokines and exhibit modest cytotoxicity along with antibody-dependent cellular cytotoxicity (ADCC) function. These cells produce INF-γ, TNF-α, GMS-CSF, and IL-10; therefore, they also modulate the T-cell response and angiogenesis. Their predominant localization is the secondary lymph tissues. (b) NK cells characterized by CD56^dim^/NKp80+/CD16+/perforin+/granzyme B+/KIR+ are capable of inducing cytotoxicity via perforin, granzyme release, and death receptor activation, exhibiting enhanced cytotoxicity and ADCC functionality. They can be found in peripheral blood to eliminate infected or malignant cells, and their terminal maturation is in the bone marrow. (c) An adaptive NK cell characterized by CD56^dim^/CD57+/NKG2C+/FcεRIγ−/PLZF− possesses an enhanced ability to release cytokines such as IFN-γ and TNF-α, with a robust response against repeat antigen exposure. They can be found in the blood, liver, and spleen and usually arise after a viral infection [4,5,8,21,26,27,28].

## 3. What Are Their Main Characteristics and How Do They Function?

**Figure 1:**
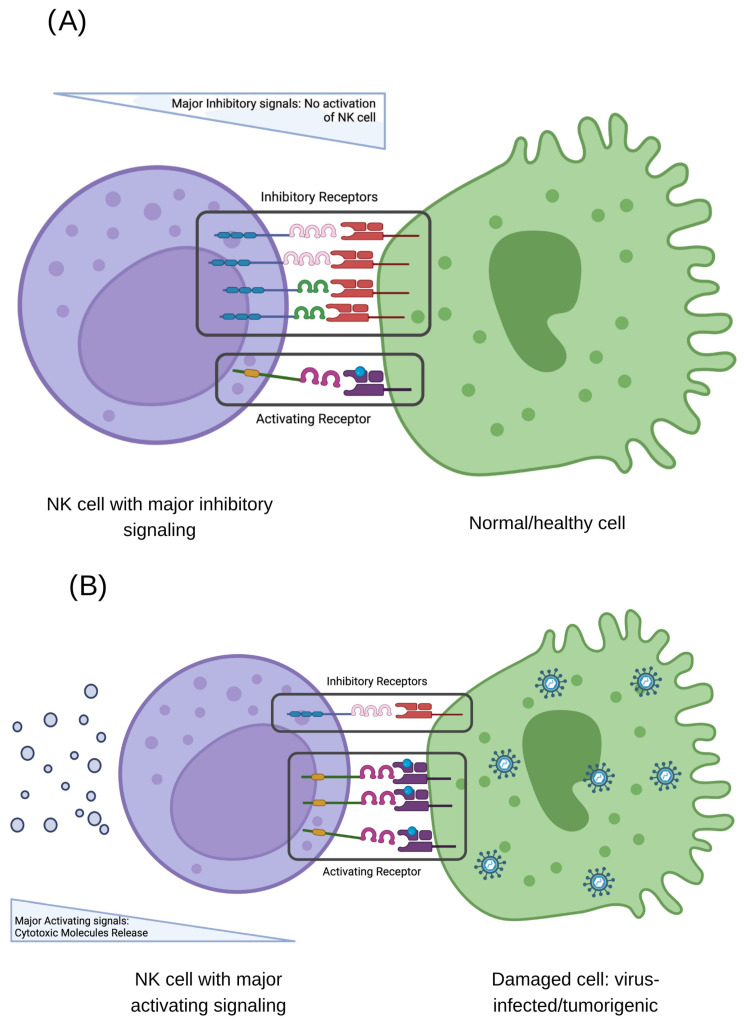
The functional activity of natural killer (NK) cells is tightly regulated by a finely balanced integration of activating and inhibitory signals, mediated through interactions between NK cell surface receptors and class I human leukocyte antigen (HLA) molecules on potential target cells. The outcome of this interaction depends on the specific repertoire and relative expression levels of these receptors. Two principal scenarios can be distinguished: (**A**) When inhibitory receptors predominate and effectively engage with their cognate HLA ligands on the target cell, a negative signal is transduced, leading to the suppression of NK cell cytotoxic activity and the preservation of the target cell, typically indicative of a healthy, non-infected state. (**B**) Conversely, if activating receptors prevail in the interaction with their respective HLA ligands, the balance shifts toward the activation of the NK cell, culminating in the targeted elimination of the infected or transformed cell through the release of cytolytic effector molecules. Created in BioRender. Alvarado Hernández, D. (2022) BioRender.com/f09s127.

The intricate mechanisms by which NK cells combat pathogens and potential threats have evolved over millennia of interactions between microorganisms and their human hosts [29]. These mechanisms have undergone evolutionary pressures to become increasingly specialized and efficient in activating or inhibiting their immune responses. Various built-in cell surface receptors play a crucial role in these strategies, mainly focused on keeping a watchful immune response against viruses. In addition to the work of these surface receptors, the class I HLA molecules are crucial because they are the main partners they interact with (Figure 1) [1,13,30,31].

**Figure 2:**
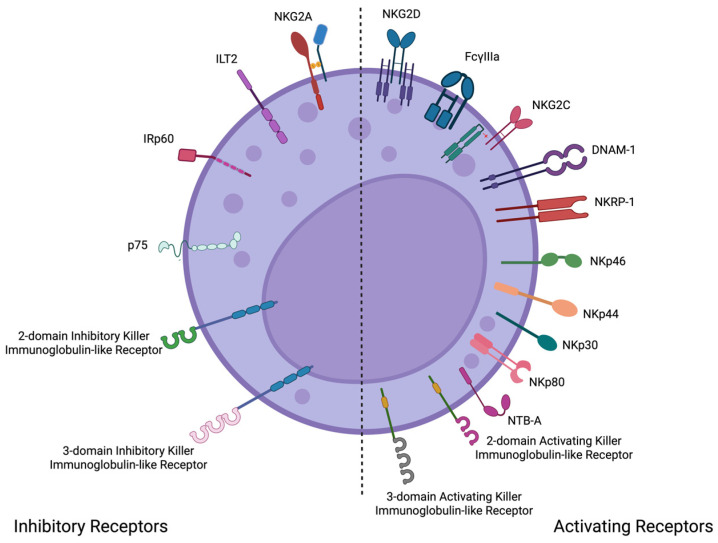
The expression of surface receptors on NK cells is a crucial aspect of their development in the bone marrow. The process could be broadly summarized into three stages of maturation, the third being the one that commits the immature NK cell to turn into a cytotoxic lytic effector lymphocyte with the appearance of CD16+ and CD56+ surface markers. These receptors can be classified as either activating or inhibitory, depending on the signals triggered by their interaction with ligands (primarily HLA-I molecules). The left side of the image shows the main inhibitory receptors, while the right side highlights some of the most relevant activating receptors. Created in BioRender. Alvarado Hernández, D. (2022) BioRender.com/c06i351.

Based on their structure, NK cells possess two distinct families of receptors: the immunoglobulin superfamily (Ig-SF) and the C-type lectin family (CL-SF). The Ig-SF has three main types of receptors: killer cell immunoglobulin-like receptors (KIRs), Leukocyte Immunoglobulin-like Receptors (LILR), and Natural Cytotoxicity Receptors (NCRs). On the other hand, the CL-SF comprises type C-like lectin receptors (Figure 2) [8,30].

**Figure 3:**
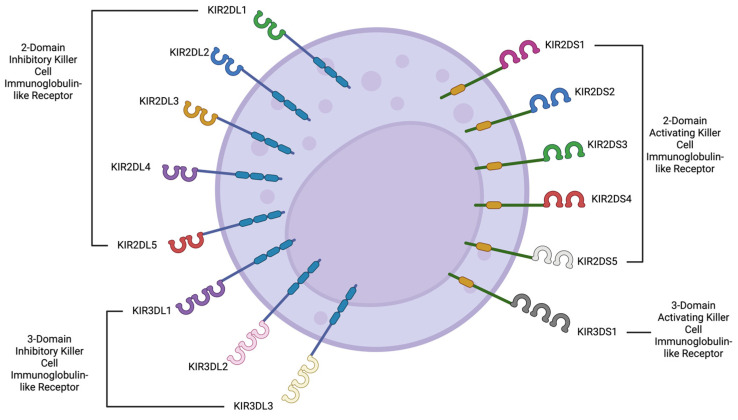
The killer cell immunoglobulin-like receptors (KIRs), members of the immunoglobulin superfamily, constitute a diverse and highly polymorphic group of surface receptors. They are classified based on the number of extracellular immunoglobulin-like domains (either two or three), the length of their cytoplasmic tail—long (L) or short (S)—and sequence homology. Structurally, KIRs with long cytoplasmic tails typically mediate inhibitory signals via immunoreceptor tyrosine-based inhibitory motifs (ITIMs), whereas those with short tails generally serve activating roles through association with adaptor molecules bearing immunoreceptor tyrosine-based activating motifs (ITAMs). These receptors are predominantly expressed on the CD56^dim^/CD16^+^ subset of NK cells, which are principally responsible for executing cytotoxic functions. Created in BioRender. Alvarado Hernández, D. (2022) BioRender.com/f00c581.

Genetically linked to the KIR gene cluster are the LILR receptors. This multi-gene family is also encoded in the leukocyte receptor complex (19q13.4). First identified in 1997, the 11 members of this family are subdivided into activating (6 receptors) and inhibitory (5 receptors) groups (Figure 3). Their expression is not exclusive to NK cells; it is also observed on the surface of antigen-presenting cells (APCs), such as dendritic cells, macrophages, and B cells. These receptors are involved in various activities, including cell migration, cell proliferation, phagocytosis (mainly in APCs), cytokine synthesis and release, chemical mediator production and secretion (mainly in NK cells), and cell death (through the Fas receptor). Therefore, they are considered an important bridge between the innate and adaptive immune responses. Similar to KIR receptors, LILR receptors exert their inhibitory mechanisms through the activation of signaling pathways triggered by, among others, the tyrosine phosphatase SHP [14,15,16].

The KIR family was the first to be discovered and is encoded on the long arm of chromosome 19 in the leukocyte receptor complex. These receptors have a broad range of activating and inhibitory surface receptors. Of the 17 genes in this family, 9 are inhibitory, 6 are activating, and 2 are pseudogenes. Either a long (inhibitory) or short (activating) intracellular tail differentiates them structurally. This group of receptors is widely expressed on NK cells with the phenotype CD56^dim^/CD16+ and is the most polymorphic of the NK receptors, accounting for 2250 alleles by the middle of 2025 [1].

Additionally, the Ig superfamily contains the NCR, a group of three related proteins known as type I transmembrane glycoproteins. This group includes three different activating receptors: NKp46 (also called NCR1 or CD335), NKp44 (also called NCR2 or CD336), and NKp30 (also called NCR3 or CD337). These receptors were discovered during the late 1990s in the same laboratory where NK cells were first described. Unlike the previously mentioned groups of receptors, NKp30 and NKp44 are encoded on chromosome 6, while NKp46 is encoded on chromosome 19 (like KIR receptors). This group of receptors plays several roles in infectious and non-infectious diseases, particularly in recognizing tumor and viral ligands. For example, NKp46 specifically recognizes the surface protein hemagglutinin of the influenza A virus. Tumor tissues can produce inflammatory cytokines and chemokines that change how NCR receptors, especially NKp44, are expressed, which results in lower levels and activity against tumor cells. Several substances from cells infected by viruses have been found that can activate these NK cell receptors, including those from dengue, Newcastle disease virus, HSV1, West Nile virus, poxviruses, herpesviruses, and influenza viruses [5,29,32,33,34,35,36].

The C-type lectin-like receptor superfamily consists of proteins that cross the cell membrane and are mostly present on most NK cell types and some CD8+ T cells. These receptors play a crucial role in both innate and adaptive immunity, exhibiting inhibitory and activating functions. This group of receptors is made up of two different proteins, CD94 and one of the seven NKG2 proteins: NKG2-A, NKG2-B, NKG2-C, NKG2-D, NKG2-E, NKG2-F, or NKG2-H. NKG2-A and NKG2-B belong to the inhibitory subgroup, while the rest exhibit activating features. The primary known human ligand is the HLA-I class molecule HLA-E, although several other exogenous ligands have also been identified [29,30].

NKG2A, B, C, E, and F receptors are expressed in NK cells, CD8+ T cells, CD4+ T cells, and NKT cells. These receptors detect HLA-E combined with a leader sequence from MHC class I, which helps them evaluate how much MHC-I is present on target cells. Similarly, NK cells and CD8+ T cells express NKG2D, which identifies MICA and MICB in stressed cells. The lectin-like inhibitory receptors CD94-NKG2A interact specifically with non-classical HLA-E molecules. Human NKG2D homodimers, for example, bind to stress-related proteins like MICA and MICB (which are linked to MHC class I), as well as to ULBPs [31,37,38].

The largest group of NK cells is known as CD56^dim^/CD16+, making up to 96% of all NK cells in the blood. These cells help fight viruses by releasing IFN-γ and are very effective at killing other cells by using substances called granzymes and perforin when triggered properly. The second group, CD56^bright^/CD16+/−, is much smaller in number and mainly works by releasing signaling molecules called cytokines, such as IFN-γ, GM-CSF, and TNF-a. In addition to these mechanisms, NK cells can act indirectly through ADCC, thereby bridging innate and adaptive immunity. In the presence of specific antibodies, CD16 elicited ADCC against infected cells [31,37,38].

In the blood, NK CD56^bright^ cells express molecules, such as NKG2A, NCR, IL-12R, IL-18R, and IFNAR. Their movement to certain areas in the body is controlled by special receptors called chemokine receptors; for instance, CXCR6 helps them reach the liver, CXCR5 leads them to lymph nodes, CLA directs them to the skin, and CXCR3 guides them to the lungs. In response to an acute viral infection, these NK cells produce cytotoxic granules, a process regulated by proteins, like mTOR, STAT1, and STAT4. In contrast, NK CD56^dim^ cells express molecules, such as NKG2C, IL-12R, IL-18R, CD16, CD2, KIR, and CD57. This process happens when receptors on NK cells connect with HLA molecules on the surface of the target cell [2,32,33,34,35,36,37,39,40,41].

The relevance of the delicate balance has already been mentioned, and the numerous groups of NK cell receptors should be maintained to preserve homeostasis. A careful match between activator and inhibitory receptors is needed to either turn on the NK cell’s ability to kill other cells and produce cytokines or to keep it inactive. The actions that NK cells take when activated by KIR receptors happen when they connect with their partners, which are MHC class I molecules (HLA-B and HLA-C). When KIR receptors activate NK cells, they do so by linking infectious and non-infectious diseases [42,43,44,45,46,47].

NK cells can exhibit either an activating or inhibiting profile. When NK cells have certain receptors like NKG2, DNAM1 (CD226), CD16 (Fcγ-RIIIA), C-type lectin, UL16, SLAM receptors, NKp46, NKp30, NKp44, and KIRs 2DL4, 2DS1, 2DS2, 2DS3, 2DS4, and 3DS1, they are active and can kill other cells. On the other hand, when NK cells have inhibitory receptors such as KIRs 2DL1, 2DL2, 2DL3, 2DL5, 3DL1, and NKG2A, they show an inhibitory behavior, identifying and protecting healthy cells [48].

The above-mentioned information about the characteristics and functions of NK cells is essential to determining their importance and role during SARS-CoV-2 infection. For example, NK cells participate in the early containment of the SARS-CoV-2 virus by detecting and killing infected respiratory epithelial cells. The recognition of the viral infection is driven by the NKp30, NKp46, and NKG2D receptors [5]. Another mechanism is the ADCC elimination drive by CD16 [49]. On the other hand, the virus reduces the NK cell count, particularly the CD56^dim^ subset, which could be interpreted as a functional exhaustion. It has been reported that in severe cases, there is an increased expression of the inhibitory receptor NKG2A [50]. In the next section, we will explain the role of NK cells in other infections as an example of their relevance.

## 4. The Fight of NK Cells Against Pathogens

As mentioned earlier, NK cells help fight not just viral infections but also certain parasites and bacteria, like the one that causes malaria, *Plasmodium falciparum.* When this parasite is around, the immune system kicks into action, and NK cells make interferon-γ, which prevents the parasite from growing and leads to the death of infected red blood cells, as well as activating a process that helps destroy infected cells with the help of antibodies. One possible reason for this difference is whether or not certain KIR receptors are present, with haplotype B (which mostly has inhibitory receptors) being more common in some people (Table 1) [51].

Another example of the role of NK cells during a parasitic infection is toxoplasmosis, caused by the intracellular parasite Toxoplasma gondii. These immune cells (together with CD4+ and CD8+ T cells) are activated by the proinflammatory cytokine IL-12, which then triggers the release of IFN-γ, a key and powerful part of the body’s first line of defense against viral infections. A study conducted in Brazil found that specific KIR genes, like KIR2DL2 and KIR2DS2, affect how well people are protected from developing eye infections caused by toxoplasmosis [54] (Table 1).

Intracellular bacteria such as rickettsia and chlamydia can also induce NK cell activities. For example, the release of IFN-γ, along with help from other cells and cytokines in the adaptive immune system, is crucial for managing and getting rid of these bacteria from infected areas, like the eyes, lungs, and genital organs. While NK cells are not as directly involved in the withdrawal of pathogens as macrophages are, they are potent inducers of macrophage polarization from M1 to M2 profiles. Additionally, NK cells are involved in the immunomodulation of dendritic cells, indirectly promoting a Th1 polarization [12,55].

One of the most significant viral events of the 21st century was the influenza A/H1N1pmd2009 pandemic. During this pandemic, it was shown that the way HA viral proteins connect with NCR receptors on NK cells is crucial for how the disease develops. Ideally, this interaction triggers the lysis of infected cells. However, a change in the sugar coating of HA proteins—used by the virus to escape detection—can prevent the interaction between the receptors and viral proteins, making it harder for the immune system to respond [4,8,56] (Table 1).

Researchers have also found that the activity of NK cells and their receptors influences the Ebola virus infection. In a 2010 case-control study, researchers examined the expression of KIR genes in groups of individuals who either survived or died from Ebola virus infection, as well as those who had never been infected. They found that the presence of two activating KIR genes was more frequently observed in individuals who succumbed to the infection. On the other hand, in diseases caused by the hepatitis C virus, having more inhibitory NK cell receptors like KIR2DL3, KIR2DL5, and KIR2DL3 was linked to a quicker move to the chronic stage of the disease [42] (Table 1).

Finally, the study determined the influence of KIR gene frequency in a Mexican population of pregnant women infected by cytomegalovirus before or during pregnancy. It was hypothesized that an increased frequency of activating genes would likely prevent infection by this highly distributed virus. Importantly, it was concluded that it is not a single gene responsible for the protective effect; on the contrary, specific combinations given by the position of the genes in the chromosome (either centromeric or telomeric) were shown to be related to the protective effect regarding the pathogenicity of this virus [53].

## 5. NK Fights Against SARS-CoV-2

Understanding the immune system’s response during SARS-CoV-2 infection remains a topic with many gaps yet to be filled. The wide variety of clinical presentations observed due to SARS-CoV-2 has become a hallmark of this recently discovered coronavirus. While 20–40% of infected patients remain asymptomatic, the majority develop a mild infection, and only 10–20% require hospitalization and intensive care. Researchers have attributed differences among these groups to factors such as genetics, comorbidities, age, and the immune system. The innate immune system’s role in this viral infection’s progression is undeniable, with NK cells playing a significant part. NK cells maintain their well-known mechanisms of cytotoxicity and proinflammatory cytokine synthesis, which greatly influence the immune response to SARS-CoV-2 [4,48]. Under conditions that are still not fully understood, these mechanisms can become exacerbated and dysregulated, leading to the cytokine storm, which is often associated with increased mortality. We have observed this abnormal response even in patients without any underlying comorbidities or risk factors [57].

In patients with severe SARS-CoV-2 infection, there has been a drop in the number and effectiveness of NK cells, along with an increase in certain inhibitory receptors, like KIR and lectin C-type. The cytokine storm can result in respiratory distress syndrome, as shown in Table 2. Additionally, having fewer NK cells and a higher CD56^dim^/CD56^bright^ ratio is linked to a worse outlook for recovery [2,58,59]. In this regard, the decrease in the number, along with an increased CD56^dim^/CD56^bright^ ratio, is associated with a worse prognosis. Additionally, studies have shown that a normal NK cell profile returns to normal levels once the patient recovers [59,60].

A retrospective study involving 168 infected patients, categorized into severe and non-severe cases, highlighted the role of NK cells during SARS-CoV-2 infection. An association was observed between the capacity to clear viral RNA and an increase in the number of circulating NK and T lymphocytes. Conversely, patients exhibiting NK lymphocytopenia had a worse prognosis and decreased survival rate [61]. The influence of comorbidities such as obesity and hypertension on the progression to severe COVID-19 has been consistently observed. A study of 156 Brazilian patients with molecularly diagnosed COVID-19 and hypertension revealed an increased expression of the NKG2A lectin receptor in those who progressed to severe disease. The primary role of this receptor is to inhibit the cytotoxic activities of NK cells, which are crucial for controlling viral infections [62,63,64,65,66,67,68].

### 5.1. Activation, Functional Dynamics, and Exhaustion of NK Cells in SARS-CoV-2

Upon SARS-CoV-2 infection, a pronounced activation of NK cells has been documented. This activation is characterized by the upregulation of HLA-DR, CD69, perforin, and Ki-67 markers, indicating heightened cytotoxic potential and proliferative status. Studies have demonstrated that NK cells can directly recognize and lyse SARS-CoV-2-infected cells, thereby contributing to the containment of viral replication. The mechanism involves the engagement of activating receptors on NK cells with stress-induced ligands expressed on infected cells, leading to targeted cytotoxicity. Furthermore, NK cells secrete a repertoire of cytokines, including IFN-γ and TNF-α, which bolster the antiviral response by modulating the activity of other immune cells. This effect has been observed, especially when groups of different severity have been compared, specifically in patients with severe forms of the disease who express higher IFN-γ levels. However, the magnitude and efficacy of NK cell responses can vary among individuals, influenced by factors such as age, comorbidities, and genetic predispositions. On the other hand, SARS-CoV-2 infection induces notable changes in the phenotype and subset distribution of NK cells. A significant observation is the expansion of NK cell subsets exhibiting diminished cytolytic activity, coupled with an increased expression of inhibitory receptors, particularly KIR2DL1. This phenotypic shift is particularly pronounced in severe COVID-19 cases and is often associated with the presence of the C2 ligand, suggesting a ligand–receptor interaction that may attenuate NK cell function. Single-cell RNA sequencing analyses have further delineated the emergence of adaptive NK cell subsets during acute infection, characterized by distinct transcriptional profiles and receptor expression patterns. These adaptive subsets may arise as a compensatory mechanism in response to persistent viral antigens, yet their exact role in disease modulation remains an area of active investigation [63,65].

In the milieu of severe COVID-19, NK cells often exhibit signs of functional exhaustion. This state is defined by a marked reduction in the number of cells during the first two months post-recovery, reduced cytotoxic capabilities, decreased cytokine production, and sustained expression of inhibitory receptors, such as NKG2A and PD-1. The upregulation of HLA-E on infected cells, interacting with NKG2A, delivers inhibitory signals that dampen NK cell activity, thereby facilitating viral persistence. Concurrently, the hyperinflammatory environment characteristic of severe COVID-19 can further impair NK cell function, as elevated levels of proinflammatory cytokines may induce a state of chronic activation leading to exhaustion. This paradoxical scenario underscores the delicate balance between effective antiviral responses and immune-mediated tissue damage [63,64,65,66,67,68].

### 5.2. Impact of SARS-CoV-2 Variants on NK Cell Responses

Several SARS-CoV-2 variants emerged during at least the first two years of the health emergency, exhibiting varying degrees of pathogenicity and infectivity, mainly due to frequent errors in genome replication. Variants such as Alpha (1.1.7), Beta (B.1.3.5), Delta (B.1.617.2), Gamma (B1.1.28), and Omicron (B1.1.529) showed different degrees of transmissibility and severity in symptomatology, with Delta and Omicron being some of the most infectious. The variability emerged due to several mutations located mainly in the region codifying the spike protein (S-protein), triggering a high variability regarding infectivity, incubation period, and response to vaccination. For instance, the variant of concern Omicron (B.1.1.529), which emerged by the end of 2021, exhibited a high contagious rate, a decreased incubation time, mild symptomatology, and a reduced number of severe cases. The Delta variant, on the other hand, was characterized by a high transmissibility (up to 40–60% higher than the Alpha variant); an increase in the severity of the symptoms, especially for elderly people; and a loss of sensitivity to neutralizing antibodies [64,69,70,71,72].

NK cells, which traditionally recognize infected cells through stress-induced ligands rather than specific viral epitopes, might retain their functionality against these variants. However, alterations in the viral genome could influence the expression of ligands on infected cells, potentially modulating NK cell recognition and response. Ongoing research aims to elucidate the extent to which different variants affect NK cell-mediated immunity and to identify potential strategies to enhance NK cell responses against a broad spectrum of viral mutation [68].

In another study with 32 patients (17 that survived and 15 deceased) performed in Italy, they found that compared to healthy donors, SARS-CoV-2 patients had lower levels of NK56+ cells and NK56^bright^ cells. The infected patients showed higher amounts of CD69, TIM-3, PD-1, and Aiolos but lower amounts of NKG2D, Siglec-7, DNAM-1, and CXCR6. When they compared the levels of NK cells between the survivors and the deceased, those who survived showed higher levels of bright and adaptive NK cells. The authors conclude that in COVID-19 patients, there was a defective function of the NK cells [73].

Researchers postulate that the disease disrupts the essential role of NK cells in controlling SARS-CoV-2 infection. Additionally, low levels of circulating NK cells have been reported to correlate with severe COVID-19 cases [31,54,71]. The peripheral reduction in NK cells may be explained by their migration to the lungs. High expression levels of HLA-E (ligand for NKG2C and NKG2A) in the respiratory epithelium stimulate the cytotoxic activity of NKG2C+ NK cells against SARS-CoV-2-infected cells, while NKG2A+ inhibits NK activity. In patients with severe COVID-19, characterized by a cytokine storm, an increase in the expression of NKG2A+ NK cells and a reduction in NKG2C+ NK cells have been observed. These findings suggest the presence of exhausted NK cells, a factor associated with lung damage [48,63,74,75,76].

In patients with severe SARS-CoV-2 infection, the accumulation of cytokines such as TGF-β, TNF-α, IL-1, IL-2, IL-5, IL-6, IL-8, IL-10, and IL-18 may impede viral clearance. The cytokine storm is a clear indication of the immune system’s deviation from an effective response to infection. In part, this phenomenon is caused by alveolar macrophages, which are immune cells specialized in recognizing foreign antigens through pattern recognition receptors (PRRs), such as Toll-like receptors (TLRs). During SARS-CoV-2 infection, the virus activates various immune cells, including these macrophages. Upon engagement of TLRs on macrophages by SARS-CoV-2 components, these cells initiate the secretion of proinflammatory cytokines, notably (but not exclusively) tumor necrosis factor (TNF) and interleukin-1 (IL-1). These macrophage-derived cytokines subsequently activate additional immune cell populations, resulting in increased vascular permeability, pulmonary edema, fluid extravasation, apoptosis, and endothelial damage. Collectively, these events contribute to the onset and propagation of systemic inflammation. Indeed, type 2 pneumocytes, which express the ACE2 receptor, are also involved in this process, facilitating the host’s response to the infection. The binding of SARS-CoV-2 to the ACE2 receptor elicits the production of inflammatory cytokines. In the case of respiratory viruses, particularly SARS-CoV-2, these deviations cause inflammatory damage to the lungs. In patients with severe COVID-19, high levels of IL-6 and TGF-β have been detected. These patterns of cytokines lead to a condition called macrophage activation syndrome, which results in severe lung inflammation and reduces the effectiveness of NK cells. This hypothesis is one of the rational ideas behind the use of tocilizumab, an anti-IL-6R monoclonal antibody, as well as Leronlimab, a CCR5 antagonist monoclonal antibody [3,4,48,75,77,78].

The phenotypic profile of NK cells in non-severe SARS-CoV-2 infection is characterized by an increase in the expression of CD69, CD38, HLA-DR, Lag-3, Tim-3, and TIGIT, with an increase in the production of perforin, MIP-1b, and granzyme B. On the other hand, the phenotypic profile of NK cells in patients with severe illness showed an increase in KIR receptors, CD57, NKG2C, and IL-15R, along with a reduction in the production of perforin and granzyme B. It has been postulated that in patients with severe COVID-19, there is a high abundance of CD56^dim^ NK cells in peripheral blood [8].

COVID-19 studies performed after death have found signs, like blocked small blood vessels, widened air passages, collapsed air sacs, a thick membrane, shedding of lung cells, and the presence of certain immune cells. All these findings can be visualized as a heavy, congested, and diffusely edematous lung parenchyma. The inflammatory infiltrate includes macrophages, CD4+ T cells, CD8+ T cells, and NK cells. This inflammatory process could lead to lung fibrosis [76].

In healthy people, activated NK cells show low amounts of Ki-67, HLA-DR, CD69, perforin, NKG2C, and Ksp37. However, in cells infected with SARS-CoV-2, the levels of these receptors and proteins go up, along with an increase in CXCR3, CXCR6, and CCR5 receptors. This increase is due to the expression of viral peptides through HLA-E, triggering the “missing-self” recognition signal detected by NK cells, which facilitates their activation. Additionally, severe COVID-19 disease outcomes have been associated with decreased peripheral NK cells and NK cell hyperactivity. The decrease in peripheral NK cells happens because they move to the lungs, which is caused by the presence of certain chemicals called chemokines, including CCL2, CCL3, CCL4, CXCL9, CXCL10, and CXCL11. As a result, the body’s fight against the virus, which includes both normal and excessive responses, can worsen tissue damage and lead to the lung problems mentioned earlier [38].

Based on all these findings, it has been proposed that the use of activated NK cells could lead to a better prognosis and higher survival rates. Currently, several clinical trials are in progress, albeit with notable discrepancies in the results. Nonetheless, this approach is considered a promising therapy [2,74].

The creation of genetically modified CAR-NK cells has become a new way to improve how well NK cells can specifically attack and kill cells infected with SARS-CoV-2. CAR-NK cells are safer than T cells because they have a lower chance of causing graft-versus-host disease (GVHD), making them a better choice for treatments using cells from donors. Several CAR constructs have been designed to target viral proteins, such as the SARS-CoV-2 spike glycoprotein, and facilitate targeted NK cell activation upon encountering infected cells. Recent studies have shown that CAR-NK cells with improved receptors, like those that include NKG2D or CD16, are better at killing infected cells and last longer in the body. Also, CAR-NK cells that can target both viral proteins and stress signals in infected cells have been shown to work better together against viruses. However, there are still challenges in using CAR-NK cell therapy, such as the need to produce them in large amounts and ensure they last a long time in the body, which need to be improved before they can be widely used in clinics [70,79,80,81,82].

### 5.3. New Insights in Cellular Therapies Using NK Cells

NK cells, as essential effectors of the innate immune system, have attracted considerable attention for their therapeutic potential in combating SARS-CoV-2 infection. Their ability to mediate cytotoxicity through perforin-granzyme release, antibody-dependent cellular cytotoxicity (ADCC), and cytokine production positions them as promising candidates for targeted immunotherapeutic strategies. Since its identification in 2020, SARS-CoV-2 infection has been treated with pharmacological therapies, such as lopinavir/ritonavir. However, the dysregulation of NK cell activity in severe COVID-19 cases, characterized by functional exhaustion, reduced cytotoxicity, and altered receptor expression profiles, necessitates interventions aimed at restoring NK cell functionality. Several approaches, including adoptive NK cell therapy, chimeric antigen receptor (CAR)-NK cells, and cytokine modulation, are currently being explored to harness and enhance the antiviral capacity of NK cells in COVID-19. One of the most direct therapeutic strategies involves the infusion of ex vivo-expanded NK cells derived from peripheral blood, umbilical cord blood, or induced pluripotent stem cells (iPSCs). This approach aims to compensate for the NK cell depletion observed in severe COVID-19 cases. Preclinical studies have demonstrated that allogeneic NK cell transfusions exhibit robust antiviral activity against SARS-CoV-2-infected cells, reducing viral loads and mitigating inflammatory responses. A notable clinical trial investigating the use of CYNK-001, an allogeneic NK cell product derived from placental CD34+ cells, has shown promising preliminary results, with treated patients exhibiting enhanced viral clearance and reduced disease severity. However, challenges remain, particularly regarding NK cell persistence and the immunosuppressive microenvironment induced by hyperinflammatory states in severe COVID-19. Strategies to optimize NK cell survival post-infusion, such as co-administration with IL-15 or genetic modifications to enhance metabolic fitness, are under investigation [79,80,81].

## 6. Conclusions

The SARS-CoV-2 pandemic, during the last 4 years, has underscored the intricate and often unpredictable nature of viral disease evolution. Despite substantial scientific efforts, the underlying causes of the vast heterogeneity in COVID-19 symptomatology and clinical outcomes—including its lethality in millions of cases—remain a major challenge to our understanding. It is widely recognized that innate immunity, and particularly natural killer (NK) cells, plays a pivotal role not only in controlling viral infection but also in shaping the development of severe disease manifestations.

As key effectors of the innate immune system, NK cells execute their antiviral functions through a sophisticated network of surface receptors. The fine-tuned balance between activating and inhibitory receptors is essential for maintaining immune homeostasis, governing both cytolytic activity and cytokine secretion. While these mechanisms have demonstrated effectiveness against various infectious diseases, the emergence of COVID-19 has exposed a profound interindividual variability in NK cell responses, influenced by factors such as comorbidities and genetic diversity, particularly the polymorphic nature and frequency of certain receptor genes, such as the KIR ones.

This review highlights both the commonalities and distinctions in NK cell-mediated mechanisms against well-characterized viral pathogens such as cytomegalovirus, Epstein–Barr virus, and hepatitis C virus, drawing contrasts with the immune response observed in SARS-CoV-2 infection [8,53,83,84]. The observed variability in the expression and frequency of NK cell-activating and -inhibitory receptors is strongly correlated with the heterogeneity in disease progression and clinical outcomes. Despite the current absence of a universally effective pharmacological treatment for COVID-19, the accumulating body of evidence regarding the central role of NK cells in antiviral immunity points toward promising therapeutic avenues. Strategies aimed at modulating or enhancing NK cell function—including the development of advanced cellular therapies such as CAR-NK cells—represent a compelling opportunity to harness their innate cytotoxic potential as “professional killers” of virally infected cells. Ongoing research in this field is essential for translating these insights into clinical interventions that may improve patient prognosis and survival.

## Figures and Tables

**Table 1:** Roles of NK cell surface receptors in viral as well as non-viral infectious processes. The activities of diverse surface receptors have been highlighted in bacterial infections such as *P. aeruginosa*; parasitic infections such as *Toxoplasma gondii*; and some mycobacteria, for instance, *M. tuberculosis* and *M. bovis*.

NK Cell Receptor Type	Main Roles on Viral Infections	Main Roles on Non-Viral Infectious Processes
Natural Cytotoxicity Receptors (NCR)	NKp46, NKp30 and NKp44 are underexpressed in patients infected with HIV [2]. NKp46 recognizes influenza hemagglutinin triggering the elimination of the infected cell [3]. NKp44 serves as a ligand to the flavivirus West Nile virus, triggering its activation and elimination of the infected cell [4].	NKp44 has been showed to serve as a direct binding site for several pathogens, such as *Mycobacterium bovis* and *tuberculosis*, as well as *Pseudomonas aeuroginosa* [4,5,8].
Leukocyte Immunoglobulin-Like Receptors (LILRs)	LILRB1: upregulation has been associated with HCMV reactivation, after lung transplantation [13].	LILRA2: augmented expression has been observed in patients suffering from leprosy [52].
Killer Immunoglobulin-like Receptors (KIRs)	KIR2DS1/2DS3 are found more frequently in the patients who died due to the Ebola virus [14]. KIR2DL3/2DL5 are found more frequently in patients infected by hepatitis C with a more rapid onset of the chronic phase of the disease. Statistically lower frequencies of the haplotype cB03|tA01, containing the inhibitory gene KIR2DL5 and the pair KIR2DS3/2DS5, were observed among the pregnant women infected with CMV [53].	KIR2DL2/2DS2: an increased frequency in patients who did not develop ocular toxoplasmosis [54].

**Table 2:** The influence of the NK cells’ surface receptors has been manifested in different studies with several populations of diverse features in patients infected with SARS-CoV-2. In the majority of the presented studies, a prevalence of activating receptors has been associated with the severe form of the disease.

Studied Population	Involved Receptors	Proposed Mechanism
27 Norwegian SARS-CoV-2-positive patients with moderate and severe forms of the disease [29]	In general, the number of NK cells observed in both the moderate and the severe groups is decreased; however, an especially activated profile either for CD56^dim^ or CD56^bright^ is also observed, responsible for the typical aggressive symptomatology due to the cytokine storm.	The NK cells found in the lung tissue show a strong immune activation and thus increased interferon response, especially in the patients with the moderate version of the disease. On the other hand, the patients with the severe form of the disease showed an increased population of neutrophils and monocytes.
424 Saudi patients SARS-CoV-2 positive with different degrees of severity ranging from asymptomatic to severe forms of the disease [30]	KIR2DS4 and KIR3DL1 were observed to be in a higher frequency in patients with the severe form of the disease. KIR3DL1, 2DL5, 2DS1, 2DS5, and 3DS1 were associated with a protective effect.	KIR2DS4 and KIR3DL1 are part of the inhibitory KIR A haplotype, which could be stopping the NK cell from interacting with their HLA ligand exert a cytotoxic mechanism against the infected cell.
100 US American patients SARS-CoV-2 positive with different degrees of severity ranging from asymptomatic to severe forms of the disease [31]	The expression of NKG2D was observed to be decreased in the studied groups with different degrees of the disease (from asymptomatic to severe).	A specific phenotypic profile was observed for the healthy controls as well as for the patients with the severe form of the disease. The observed signature describes a NK cell population with decreased cytotoxicity in the severely ill patients, which could explain the increase in the symptomatology.
28 Italian patients SARS-CoV-2 positive with different degrees of severity, and grouped according to their need of oxygen support [37]	A generalized decrease in the number of lymphocytes (including NK cells) was observed in all three categories. The phenotypic profile of the circulating NK cells was mainly inhibitory, with an increased expression of NKG2A and KIR2DL1, as well as a decrease in the number of CD16+/CD56^bright^ NK cells, mainly cytokine producers.	There seems to be an increased turnover of inflammatory NK cells in the patients who required oxygen support and mechanical ventilation, suggesting an imbalance and a lack of regulation between the cytotoxic and cytokine-producing NK cells with an activating profile.
